# Efficacy of an Oscillating Chitosan Brush Versus an Air Abrasive Device in the Management of Peri-Implant Mucositis: A Randomized Clinical Trial

**DOI:** 10.3390/jfb16100387

**Published:** 2025-10-15

**Authors:** Kerem Bahçeci, Bahattin Alper Gültekin, Serdar Yalçın

**Affiliations:** Department of Oral Implantology, Institute of Health Sciences, Istanbul University, Istanbul 34116, Turkey; alperg@istanbul.edu.tr (B.A.G.); serdar.yalcin@istanbul.edu.tr (S.Y.)

**Keywords:** chitosan, peri-implant mucositis, dental implants, air abrasion, periodontal debridement, plaque index, bleeding on probing

## Abstract

This randomized, prospective clinical trial was conducted to compare the effectiveness of the oscillating chitosan brush (OCB) and an air-abrasive device (AAD) in improving clinical outcomes during non-surgical management of peri-implant mucositis. Fifty-eight patients were randomized and received baseline treatment; of these, 50 completed the 6-month follow-up. Probing pocket depth (PPD), bleeding on probing (BoP), and plaque index (PI) were assessed at six locations per implant and measured again at 2 weeks, 4 weeks, 3 months, and 6 months post-treatment. Differences between groups and time points were analyzed using non-parametric tests (Kruskal-Wallis, Dunn, Friedman, and Fisher’s Exact Test). Both treatment groups demonstrated significant improvements in PPD, BoP, and PI at the 6-month evaluation compared to baseline (*p* < 0.05). At 24 weeks, the OCB group showed faster reductions in PPD and PI compared with the AAD group (*p* = 0.03 and *p* = 0.01, respectively), while BoP did not differ significantly (*p* = 0.41). Considering the constraints of this 6-month clinical study, the non-surgical management of peri-implant mucositis using both OCB and AAD resulted in comparable clinical outcomes. Both approaches demonstrated a consistent ability to improve clinical parameters associated with this condition.

## 1. Introduction

Peri-implant diseases, namely peri-implantitis and peri-implant mucositis, were first categorized during the First European Workshop on Periodontology, which took place in Ittingen in 1993 [1]. Peri-implant mucositis develops in previously healthy peri-implant tissues due to the accumulation of bacterial biofilm around osseointegrated implants. In human studies, a clear causal relationship has been established between biofilm formation on implant surfaces and the initiation of an inflammatory response [2,3,4,5]. Bacterial biofilm build-up around osseointegrated implants plays a major role in the progression of peri-implant mucositis. Evidence from human studies confirms a clear causal pathway, where biofilm triggers an inflammatory response in the adjacent soft tissues [6].

The prevalence of peri-implant mucositis is particularly high in the early years after implant placement. For example, data indicate that within 5 years post-surgery, 10–40% of patients may experience new occurrences of this condition [7,8,9,10]. Although peri-implantitis is a frequently encountered complication, a universally accepted treatment protocol that provides consistently reliable outcomes is yet to be established [8]. Various non-surgical therapeutic approaches and instruments have been discussed in the literature; however, the complex morphology and surface irregularities of dental implants often limit the effectiveness of mechanical debridement, making complete biofilm removal a challenge [11].

The oscillating chitosan brush (OCB), commercially available as Labrida BioClean^®^ (Labrida AS, Oslo, Norway), has been developed to facilitate biofilm removal in areas that are difficult to access around dental implants and natural teeth. Chitosan, the material used in the brush filaments, is a biocompatible and biodegradable polysaccharide obtained from chitin, and exhibits inherent antibacterial properties [12].

AADs represent another minimally invasive modality for biofilm removal in peri-implant therapy. These systems typically use a stream of compressed air mixed with abrasive powders, such as glycine or erythritol, which are less aggressive compared to conventional sodium bicarbonate powders. The fine particle size and low abrasiveness of these powders allow for effective disruption of the subgingival biofilm while minimizing the risk of damage to implant surfaces and surrounding soft tissues. Clinical studies have demonstrated that AADs can reduce bleeding on probing and inflammatory signs in peri-implant mucositis, with favorable patient-reported outcomes such as reduced discomfort during treatment [13,14]. Furthermore, AADs can access narrow peri-implant spaces and provide efficient debridement, supporting their role as a safe and biocompatible adjunct in the non-surgical management of peri-implant diseases [15]. While both oscillating chitosan brushes and air-abrasive devices have been individually investigated in peri-implant therapy, there is limited evidence directly comparing these two modalities. The present study therefore provides novel clinical data by evaluating their relative efficacy in a randomized design. Recent systematic reviews and meta-analyses have highlighted the need for high-quality comparative trials in this field [15,16,17].

This randomized controlled clinical trial aimed to evaluate the impact of oscillating chitosan brush (OCB; test group) and air-abrasive device (AAD; control group) debridement on clinical outcomes such as probing pocket depth (PPD), bleeding on probing (BoP), and plaque index (PI), assessed at 2, 4, 12, and 24 weeks post-treatment. These interventions were chosen due to their minimally invasive and biocompatible nature, as well as their established use in the non-surgical management of peri-implant mucositis, whereas traditional instruments such as metal curettes or ultrasonic scalers may cause surface damage or soft tissue injury.

The null hypothesis of this study was that there would be no significant difference in the clinical outcomes (PPD, BoP, and PI) between the OCB and AAD groups in the non-surgical management of peri-implant mucositis.

## 2. Materials and Methods

### 2.1. Trial Design and Methodology

This study was conducted as a two-arm, parallel-group randomized controlled trial, with stratification by implant diameter (mini vs. regular) performed prior to randomization to ensure balanced allocation. The condition of peri-implant mucositis was defined by a PPD ranging from 3 to 5 mm, a BoP score of at least 1, and the absence of radiographic evidence of bone loss [11,18,19]. Participants were stratified based on implant diameter into regular and mini-implant categories and subsequently randomized into either the test group, which received OCB treatment, or the control group, which underwent debridement with AAD. Only one implant per patient was included. In cases where more than one implant met the inclusion criteria, a single implant was randomly selected by lot to avoid allocation bias.

Blinding was performed at the patient level; participants were not informed about which intervention (OCB or AAD) they received. Although they could observe that an instrument was applied during the procedure, they were unable to recognize the type of device or treatment principle and thus remained blinded to the allocated intervention. However, due to the nature of the procedures, the operator performing the interventions could not be blinded. The outcome assessments were conducted without disclosing the allocated intervention to the patients.

The trial adhered to the principles of Good Clinical Practice and the Declaration of Helsinki. This randomized clinical trial was designed, conducted, and reported in accordance with the CONSORT 2010 guidelines for randomized controlled trials. Approval was obtained from the Clinical Research Ethics Committee of the Faculty of Dentistry, Istanbul University, Türkiye (approval number 2023/37Rev1, dated 19 December 2023). Further authorization was granted by the Turkish Medicines and Medical Devices Agency (application number E-85521274-000-2837317, dated 29 December 2023). The study was prospectively registered at ClinicalTrials.gov (NCT06287957), and written informed consent was obtained from all the participants.

### 2.2. Sample Size Assessment and Power

Sample size estimation was performed using PPD as the primary endpoint. A clinically meaningful difference of 1 mm between the test and control groups was anticipated, with a standard deviation of 0.8 mm adopted from prior studies [20]; at a significance level of 0.05 and a statistical power of 95%, the required minimum sample size was 12 patients per arm. Considering an anticipated attrition rate of approximately 10% over the 24-week observation period, the total enrollment target was set at 50 patients.

### 2.3. Participant Selection and Characteristics

During the period from January 2024 to December 2024, consecutive individuals presenting either for supportive peri-implant maintenance or referred specifically for peri-implant mucositis were assessed for potential participation. A total of 58 patients underwent eligibility screening at the Department of Oral Implantology, Istanbul University. The primary inclusion criteria were as follows: (i) patients who had BoP, which was defined as a minimum score of 1 (i.e., punctate bleeding); (ii) those aged >18 years; (iii) those who had adequate psychological capacity to participate; and (iv) those with at least one implant located in the maxillary or mandibular posterior region (premolar or molar area, corresponding to teeth numbered 4, 5, 6, or 7) restored with a cement-retained prosthesis not splinted to other implants or teeth.

The exclusion criteria were as follows: (i) pregnant women; (ii) those with a history of, or ongoing, chemotherapy and/or radiotherapy; (iii) those with peri-implant bone loss; (iv) those with implants adjacent to the target implant; (v) those with prostheses splinted to other implants or natural teeth; (vi) those with screw-retained prostheses; and (vii) those with uncontrolled diabetes mellitus.

### 2.4. Randomization and Allocation

Participants were initially stratified based on implant platform size into either the mini or regular implant group. Following stratification, subjects were randomly assigned to treatment arms using computer-generated block randomization (RANDOM.ORG, Randomness and Integrity Services Ltd., Dublin, Ireland) under the supervision of the study coordinator. Interventions were administered in accordance with the assigned group allocation.

### 2.5. Evaluated Clinical Parameters and Outcome Measures

The evaluated variables were PPD, BoP, PI, and keratinized mucosa (KM) width. Baseline and follow-up measurements (2, 4, 12, and 24 weeks) were performed for PPD, BoP, and PI, whereas KM was assessed only at baseline. The width of KM was measured from the mucogingival junction to the free gingival margin at the mid-buccal aspect of each implant site, using HuFredy Williams periodontal probes. Each implant was examined at six reference sites: mesiobuccal, buccal, distobuccal, distopalatal/distolingual, palatal/lingual, and mesiopalatal/mesiolingual. Peri-implant inflammation was graded with the BoP index [0 = no bleeding; 1 = isolated bleeding points(punctate bleeding); 2 = continuous red line(linear bleeding); and 3 = profuse bleeding(distinct bleeding)], according to Roos-Jansåker et al. [21] PPD was recorded in millimeters, and plaque accumulation was evaluated using the Plaque Index (PI) on a 0–3 ordinal scale, as previously described [22]. The scoring criteria were: 0 = no plaque; 1 = plaque detectable only by probing; 2 = visible plaque; 3 = abundant plaque. The assessment was carried out by gentle probing of the implant collar at six sites per implant.

Clinical measurements were performed using Williams periodontal probes (Hu-Friedy, Chicago, IL, USA) under gentle pressure. All measurements were performed by a single experienced examiner (K.B.), trained in oral implantology, to ensure consistency across time points. Implant-supported prostheses were not removed during examinations.

For PPD, sites exhibiting probing depths of ≥3 mm were identified individually and monitored over time.

Changes in the PPD and BoP were designated as the primary outcome parameters, with PI serving as a secondary outcome parameter.

Adverse events and complications were monitored at each follow-up visit through clinical examination and patient self-reporting. No adverse events were recorded during the study period.

### 2.6. Description of Interventions

Patients assigned to the test group underwent implant debridement using OCB. Labrida BioClean brushes were immersed in sterile saline for a minimum of 2 min, as per the manufacturer’s guidelines, and subsequently mounted on a Woodpecker Endo PACE motor (Guilin Woodpecker Medical Instrument Co., Ltd., Guilin, China). The brush was gently maneuvered within the peri-implant sulcus with light sweeping movements for a total of 2 min. The sulcus was then rinsed thoroughly with sterile saline.

In the control group, treatment was performed using the EMS Handy 3.0 Perio Premium device (Electro Medical Systems S.A., Nyon, Switzerland). The device’s reservoir was filled with glycine powder, and Perio-Flow nozzles were attached to the instrument. The subgingival nozzle was inserted into the peri-implant sulcus, and circumferential debridement around the implant was performed for a total duration of 2 min. Upon completion, the sulcus was irrigated with sterile saline.

Both treatment protocols were implemented once at baseline, and all patients were followed up over a 6-month period. No specific oral hygiene instruction was provided; patients were advised to maintain their usual daily oral care routine.

### 2.7. Statistical Methods and Data Processing

Clinical data were recorded in both paper files and electronic databases. Statistical analyses were carried out using R software, version 4.3.2 (The R Foundation for Statistical Computing, Indianapolis, IN, USA). Normality of distribution was verified with the Shapiro–Wilk test. When non-normal distributions were identified and three or more groups were compared, the Kruskal-Wallis H test was employed, followed by Dunn’s test for post hoc pairwise analysis. Categorical variables were analyzed using Fisher’s Exact Test combined with Monte Carlo simulation, while Bonferroni-adjusted Z tests were applied for multiple comparisons. For longitudinal non-parametric data, the Friedman test was used, with Dunn’s test for subsequent comparisons. Continuous variables were summarized as mean ± SD or median (minimum–maximum), and categorical variables as counts and percentages. Statistical significance was set at *p* < 0.05.

Specifically, PPD values were analyzed as continuous variables (median, IQR) and compared both between and within groups using the Kruskal-Wallis and Friedman tests, respectively. BoP was analyzed as an ordinal variable (0–3 categories) and presented as percentages, with intergroup comparisons performed using Fisher’s Exact Test. PI was analyzed on an ordinal scale (0–3), reported as medians and interquartile ranges, and compared with the Kruskal-Wallis and Friedman tests. KM width was assessed only at baseline as a categorical variable (none, 1–3 mm, >3 mm) and compared between groups using Fisher’s Exact Test. Analyses followed a per-protocol approach including the 50 patients who completed the 24-week follow-up; missing data from the 8 patients lost to follow-up were not imputed.

## 3. Results

A total of 58 patients were randomized and received baseline treatment. During the follow-up period, 8 patients were lost, resulting in 50 patients completing the study. No adverse events were reported. Baseline demographic and clinical characteristics of the participants are presented in Table 1. At baseline, there were no significant differences between the groups in terms of age, implant characteristics, smoking habits, or keratinized mucosa (all *p* > 0.05). Sex distribution differed significantly across groups (Fisher’s Exact Test, *p* = 0.002).

For clinical parameters, both groups demonstrated reductions in probing pocket depth (PPD), bleeding on probing (BoP), and plaque index (PI) over the 24-week follow-up. At baseline, there were no between-group differences for the clinical parameters (PPD, BoP, PI) (all *p* > 0.05). At 24 weeks, the OCB group exhibited significantly faster reductions in PPD (Kruskal-Wallis test, *p* = 0.03) and PI (*p* = 0.01) compared with the AAD group, while no significant between-group differences were observed for BoP (*p* = 0.41) (Table 2).

Full results for all time points and sites (mesial, buccal, distal, and palatal/lingual) are presented in Tables S1–S5.

### 3.1. Comparative Analysis of the PPD Changes

Tables S1–S5 summarize the intergroup differences in the PPD across all the evaluation time points. At baseline, no significant differences were observed among all the groups (all *p* > 0.05). At 2 and 4 weeks, both Mini-Labrida and Regular-Labrida demonstrated significantly lower PPD values compared with Mini-EMS and Regular-EMS (*p* < 0.001), whereas no significant differences were observed between Mini- and Regular-Labrida or between Mini- and Regular-EMS. At 12 and 24 weeks, no significant differences were observed among the four subgroups (Mini-Labrida, Regular-Labrida, Mini-EMS, Regular-EMS). However, when comparing the two main groups (OCB vs. AAD), PPD and PI were lower in the OCB group at 24 weeks (*p* = 0.03 and *p* = 0.01).

### 3.2. Intergroup Comparison of BoP and PI Values

Analysis of BoP values at baseline and during each follow-up visit revealed significant differences within each group across the different time points.

Similarly, evaluation of PI values at baseline and at each follow-up visit revealed significant differences within each group across the different time points.

### 3.3. Composite Outcome

By 6 months, each implant improved in at least one parameter. The proportion of sites showing any improvement was similar between groups; however, the OCB group achieved earlier reductions in PPD and PI. (Table 2).

### 3.4. Keratinized Tissue

At baseline, all 58 randomized patients were evaluated for KM. Among them, KM was >3 mm in 14 patients and 1–3 mm in 42 patients and was absent in 2 patients. These distributions did not differ significantly between the treatment groups (*p* > 0.05).

### 3.5. Participant Attrition

**Figure 1:**
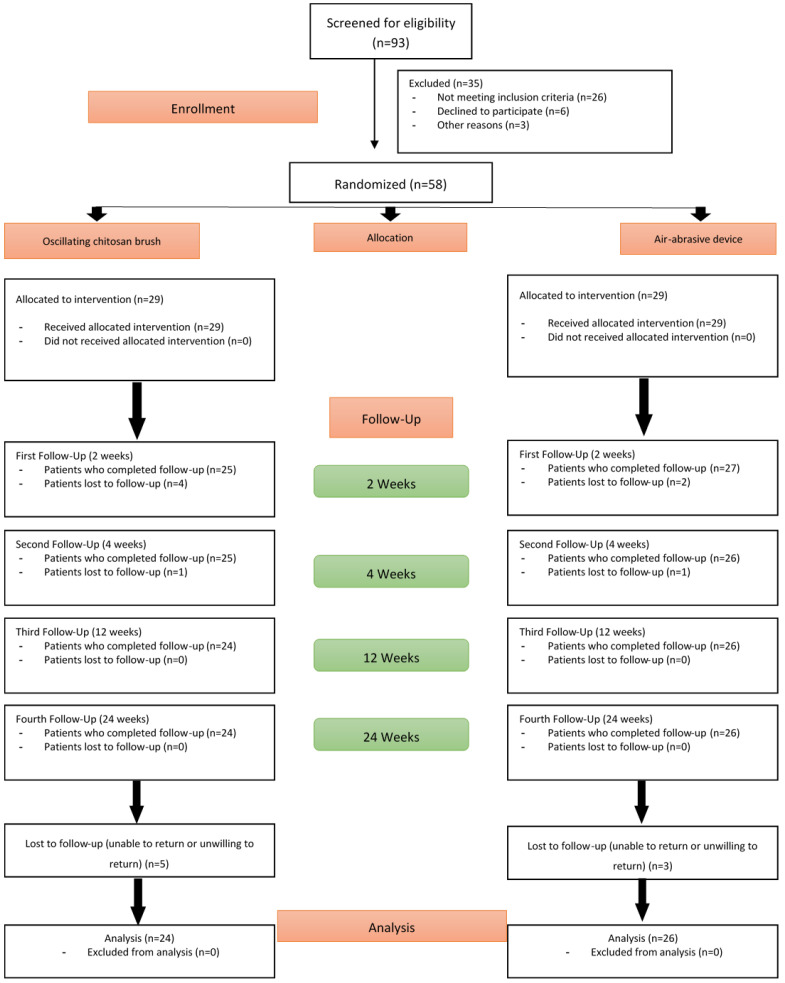
CONSORT flow diagram of participant enrollment, allocation, and follow-up.

Of the 58 patients randomized and treated at baseline, 8 patients (5 in the OCB group and 3 in the AAD group) did not attend subsequent follow-up visits. Consequently, 50 patients (24 in the OCB group and 26 in the AAD group) completed the 24-week study, as illustrated in Figure 1.

## 4. Discussion

In this prospective randomized clinical trial, the comparative efficacy of OCB and AAD debridement in peri-implant mucositis was investigated. Although no statistically significant intergroup differences were observed, both treatment modalities yielded significant reductions in the clinical signs of inflammation. These results may partly reflect the limited sample size as well as behavioral modifications and heightened awareness among participants. Each intervention was performed only at baseline, with BoP, PPD, and PI recorded at 2, 4, 12, and 24 weeks. By the 6-month follow-up, significant improvements were noted in both groups.

In a related randomized trial, 24 peri-implant mucositis sites in 11 patients were randomly assigned to treatment using a chitosan brush or a titanium curette. Clinical follow-ups were performed at 2, 4, and 24 weeks. While the chitosan brush demonstrated faster initial improvement, statistical analysis revealed no significant intergroup differences at the 6-month evaluation [20,23]. In a separate investigation including 37 patients, glycine powder air-abrasive therapy was compared with ultrasonic debridement for implants diagnosed with peri-implant mucositis. Follow-up at 3 and 6 months demonstrated that both approaches were similarly effective for non-surgical management [24]. A randomized clinical trial conducted in 2024 investigated the effectiveness of glycine-based air polishing as an adjunctive therapy for peri-implant mucositis. Patients were evaluated at baseline and at 1, 3, and 6 weeks, as well as at 3 and 6 months. Clinical outcomes, including probing depth, modified sulcus bleeding index, and modified plaque index, demonstrated significant improvement following treatment. The intervention was well tolerated, with no adverse effects reported on peri-implant tissues or implant surfaces. These findings indicate that glycine powder air-abrasive therapy offers a minimally invasive and clinically advantageous approach, emphasizing the role of early mechanical biofilm disruption in reducing disease progression toward peri-implantitis [13].

The efficacy of the OCB, as a new tool for debriding implant surfaces in the management of peri-implant mucositis has been demonstrated [25]. In a case report with a 1-year follow-up, the use of an OCB was associated with marked improvements in clinical indicators of inflammation [20]. At the 6-month follow-up, the treatment modalities demonstrated a partial decrease in peri-implant inflammation [26]. In a clinical study including 37 patients, Riben-Grundstrom et al. evaluated the outcomes of glycine powder air-abrasive therapy versus ultrasonic debridement in implants affected by peri-implant mucositis. Follow-up assessments at 3 and 6 months indicated that both approaches were suitable for non-surgical management [24].

Currently, limited clinical research directly examines the impact of implant diameter on treatment outcomes in peri-implant mucositis. Although a few studies have explored potential associations between implant diameter and peri-implant soft tissue health, the available evidence indicates no significant differences in clinical indicators such as PI, sulcus bleeding index, or PPD among implants of different diameters. Both narrow and standard-diameter implants appear to exhibit similar inflammatory responses and plaque accumulation. However, no randomized controlled trials to date have specifically evaluated whether implant diameter influences clinical response to non-surgical therapy for peri-implant mucositis. Further studies are needed to determine whether implant geometry, and diameter in particular, affects the efficacy of mechanical debridement in managing this condition [27].

The present study offers notable methodological strengths. Its randomized and prospective design provides a reliable framework for minimizing selection bias and enables dynamic observation of treatment response over time. The consistent use of clinical indicators, such as PD, BoP, and PI, at multiple follow-up intervals adds robustness to the clinical data. Additionally, having a single clinician perform all procedures under a standardized protocol helped reduce inter-operator variability, enhancing the reproducibility of the findings.

Beyond the core outcomes, several unique aspects of the present study deserve particular attention. This study offers several distinctive methodological and clinical observations that are not commonly addressed in existing literature on peri-implant mucositis. First, debridement was performed as a single-session intervention, and patients were monitored over a 6-month period without additional mechanical therapy, allowing for a clearer assessment of the long-term effects of each modality. Unlike previous studies, patients were stratified according to implant diameter (mini vs. regular) prior to randomization, ensuring balanced allocation across groups. Furthermore, strict inclusion criteria were applied, limiting the sample to cement-retained, non-splinted single implants in posterior sites, thereby reducing prosthetic variability. During the use of the OCB, partial detachment of the chitosan filaments was observed in several cases, leading to exposure of the inner metallic shaft. This mechanical failure, while not previously reported in the literature, occasionally resulted in localized patient discomfort, particularly in posterior regions where visibility and access are limited. Although no adverse soft-tissue reactions were noted, this finding raises concerns about the structural integrity and single-use durability of the current brush design. Future iterations of the OCB could benefit from enhanced filament anchorage mechanisms or composite filament technologies that combine chitosan with a flexible synthetic polymer to improve structural resilience without compromising biocompatibility.

The present findings indicate that both OCB and AAD are effective and minimally invasive treatment options for peri-implant mucositis. From a clinical perspective, the earlier reduction in inflammation observed in the OCB group was reflected in earlier improvements in clinical parameters compared to the AAD group. However, as no validated patient-reported outcomes were collected, this finding should be interpreted with caution. Future studies should incorporate standardized patient-reported outcome measures to confirm potential differences in symptom relief and comfort. In addition, the compact design and portability of the OCB, compared with the bulkier AAD system, suggest potential benefits in terms of chairside efficiency. Nevertheless, economic considerations should also be taken into account: while AAD units require a relatively high initial investment, they can be used repeatedly over an extended period of time; in contrast, OCBs are supplied as low-cost, single-use devices; however, their cumulative expense may surpass that of AAD in the long term. Importantly, by the end of the 6-month follow-up, both modalities achieved comparable treatment success, indicating that OCB does not provide superior long-term outcomes but may offer advantages in terms of patient comfort and handling in the short term. However, resolution of inflammation appeared earlier in the group treated with the OCB, with improvements in clinical parameters observed as early as the fourth week, whereas similar clinical benefits in the AAD group became evident only after approximately 12 weeks. This observation should be interpreted with caution, as no validated patient-reported outcomes were collected to support potential differences in symptom relief.

From a clinical handling perspective, the OCB appeared to be more practical and ergonomically favorable based on the operator’s subjective experience. Some patients informally reported greater comfort during OCB treatment compared to AAD; however, as no validated patient-reported outcome measures were collected, these observations should be considered anecdotal. Similarly, the brief remarks on economic aspects reflect general clinical impressions rather than data generated by this trial. Future studies should incorporate validated patient-reported outcome measures and formal cost-effectiveness analyses to substantiate these points.

Nonetheless, certain limitations must be acknowledged. An important limitation is that both interventions (OCB and AAD) were applied only once at baseline. In daily clinical practice, the management of peri-implant mucositis often requires repeated maintenance sessions; therefore, the external validity of our findings may be limited. First, the duration of follow-up was relatively limited, which may restrict insight into the long-term stability or relapse of peri-implant mucosal conditions. Second, although the overall sample size met statistical requirements, the study was not sufficiently powered to perform stratified analyses based on implant-related or patient-specific variables such as diameter, surface topography, or soft tissue biotype. Expanding the number of participants would allow for more detailed subgroup analyses and increase the robustness of statistical outcomes. Moreover, longer observation periods are needed to evaluate whether the initial clinical improvements observed can be maintained over time. These enhancements in study design would contribute to a more comprehensive understanding of treatment efficacy and its long-term implications in peri-implant mucositis management. It should also be noted that baseline characteristics, including keratinized mucosa width, were assessed in all 58 patients randomized and treated at baseline; however, clinical outcomes are reported only for the 50 patients who completed the 24-week follow-up. This distinction should be considered when interpreting the descriptive baseline data.

Another limitation is the absence of microbiological or biomarker analyses in our study. This precludes correlating the observed clinical improvements with underlying microbial or inflammatory changes. Recent studies have highlighted distinct microbial profiles in peri-implant mucositis compared with healthy peri-implant tissues [28,29,30,31]. Incorporating microbiological sampling in future research would allow a more comprehensive understanding of the biological mechanisms behind treatment outcomes.

Finally, while subjective feedback from patients and operators suggested that the OCB was more comfortable and easier to maneuver, these observations were not quantified using validated scales. Future studies should incorporate standardized patient-reported outcome measures and operator-based ergonomics assessments, such as VAS scores, application time, and access ratings, to objectively evaluate comfort and usability.

In addition, no formal examiner calibration or reproducibility testing (e.g., intra- or inter-examiner reliability with kappa statistics) was performed. Although all measurements were carried out by a single experienced examiner to minimize variability, the absence of calibration procedures remains a methodological limitation.

## 5. Conclusions

Within the limitations of this 6-month randomized clinical trial, both the oscillating chitosan brush and the air-abrasive device significantly reduced probing pocket depth, bleeding on probing, and plaque index in patients with peri-implant mucositis. Although both interventions were effective, the OCB group demonstrated faster reductions in PPD and PI at 24 weeks (*p* = 0.03 and *p* = 0.01), whereas BoP was similar between groups (*p* = 0.41). This finding should be interpreted with caution, as no validated patient-reported outcomes were collected. To avoid extrapolation beyond the available evidence, we have limited our conclusions strictly to the clinical parameters assessed. Future studies are needed to incorporate standardized patient-reported measures, longer follow-up periods, and microbiological or biomarker analyses to provide a more comprehensive understanding of treatment efficacy. Overall, both interventions can be considered effective non-surgical treatment options for peri-implant mucositis.

## Figures and Tables

**Table 1:** Descriptive statistics of the study population.

	N (%)	Mean ± SD	Median (Min–Max)
**Sex** Male Female Age	23 (39.7) 35 (60.3) -	- - 42.3 ± 12.5	- - 40 (26–65)
Daily cigarette consumption	-	6.3 ± 9.1	0 (0–30)
PPD-M0	-	4.34 ± 0.98	5 (1–6)
PPD-M24	-	2.58 ± 0.70	3 (1–4)
PPD-B0	-	2.42 ± 0.95	2 (1–5)
PPD-B24	-	2.02 ± 0.59	2 (1–3)
PPD-D0	-	3.98 ± 1.24	4 (1–6)
PPD-D24	-	2.52 ± 0.76	3 (1–4)
PPD-L0	-	2.46 ± 0.97	2 (1–5)
PPD-L24	-	1.94 ± 0.47	2 (1–3)
PI-0	-	1.60 ± 0.83	1.5 (0–3)
PI-24	-	0.94 ± 0.74	1 (0–2)
KM Width (mm)		-	-
None	2 (3.4)	-	-
>3 mm	14 (24.1)	-	-
1–3 mm	42 (72.4)	-	-
BoP-0		-	-
Punctate	6 (10.3)	-	-
Linear	32 (55.2)	-	-
Distinct	20 (34.5)	-	-
BoP-24		-	-
None	4 (8)	-	-
Punctate	33 (66)	-	-
Linear	13 (26)	-	-

Values are presented as Mean ± SD, Median (Min–Max), or N (%). PPD-M: probing pocket depth—mesial; PPD-B: buccal; PPD-D: distal; PPD-L: lingual; PI: plaque index; BoP: bleeding on probing; KM: keratinized mucosa.

**Table 2:** Comparison of the clinical parameters between the OCB and AAD groups at baseline and 24 weeks.

Parameter	Baseline OCB (Median, IQR)	Baseline AAD (Median, IQR)	*p*-Value	24 wk OCB (Median, IQR)	24 wk AAD (Median, IQR)	*p*-Value
PPD (mm)	4.0 (3–5)	4.0 (3–5)	0.65	2.5 (2–3)	3.0 (2–4)	0.03 *
BoP (%)	70	68	0.72	25	30	0.41
PI	2.0 (1–3)	2.0 (1–3)	0.55	1.0 (0–2)	2.0 (1–3)	0.01 *

* Median (IQR) values are shown; *p*-values derived from Kruskal-Wallis or Fisher’s Exact Tests. Values are presented as median (IQR) or %. *p*-values were obtained using the Kruskal-Wallis test for continuous variables and Fisher’s Exact Test for categorical variables. OCB: oscillating chitosan brush; AAD: air-abrasive device; PPD: probing pocket depth; BoP: bleeding on probing; PI: plaque index.

## Data Availability

The original contributions presented in the study are included in the article, further inquiries can be directed to the corresponding author.

## Supplementary Materials

The following supporting information can be downloaded at: https://www.mdpi.com/article/10.3390/jfb16100387/s1. Table S1. Comparison of mesial PPD values between groups over time. Table S2. Comparison of buccal PPD values between groups over time. Table S3. Comparison of distal PPD values between groups over time. Table S4. Comparison of lingual/palatal PPD values between groups over time. Table S5. Comparison of plaque index and bleeding on probing between groups over time.

## Abbreviations

The following abbreviations are used in this manuscript:

OCB	Oscillating chitosan brush
PPD	Pocket probing depth
BoP	Bleeding on probing
AAD	Air-abrasive device
KM	Keratinized Mucosa
PI	Plaque index
