# Supplementary material for: Efficacy of an Oscillating Chitosan Brush Versus an Air Abrasive Device in the Management of Peri-Implant Mucositis: A Randomized Clinical Trial
Source: J Funct Biomater. 2025 Oct 15;16(10):387. doi: 10.3390/jfb16100387 (PMC12564997; doi:10.3390/jfb16100387)
Supplement: Supplementary file 1 [file jfb-16-00387-s001.zip › jfb-3904292-supplementary.pdf]

Supplementary Material

This file contains Supplementary Tables S1–S5 referenced in the main text.

Table S1. Comparison of mesial PPD values between groups over time.

Time point	Mini-Labrida	Regular-Labrida	Mini-EMS	Regular-EMS	Test statistic	p-value
Baseline	4.5 (3–6)	4.0 (3–5)	5.0 (2–5)	5.0 (1–5)	1.616	0.656
2 weeks	3.0 (2–4)	3.0 (2–3)	5.0 (2–5)	5.0 (1–5)	23.053	<0.001*
4 weeks	2.5 (2–4)	2.0 (2–3)	5.0 (1–5)	5.0 (1–5)	21.806	<0.001*
12 weeks	2.5 (2–4)	2.0 (2–3)	3.0 (1–3)	3.0 (1–4)	7.139	0.068
24 weeks	2.5 (2–4)	2.0 (2–3)	3.0 (1–3)	3.0 (1–4)	1.984	0.576

*Between-group differences tested using Kruskal–Wallis H test; within-group comparisons performed using Friedman test. *p < 0.05 considered significant. PPD: pocket probing depth

Table S2. Comparison of buccal PPD values between groups over time.

Time point	Mini-Labrida	Regular-Labrida	Mini-EMS	Regular-EMS	Test statistic	p-value
Baseline	2.5 (2–4)	2.0 (2–5)	2.0 (1–5)	2.0 (1–3)	6.495	0.090
2 weeks	2.0 (1–3)	2.0 (2–3)	2.0 (1–5)	2.0 (1–3)	1.138	0.768
4 weeks	2.0 (1–3)	2.0 (1–3)	2.0 (1–5)	2.0 (1–3)	0.482	0.923
12 weeks	2.0 (1–3)	2.0 (1–3)	2.0 (1–3)	2.0 (1–3)	0.630	0.890
24 weeks	2.0 (1–3)	2.0 (1–3)	2.0 (1–3)	2.0 (1–3)	0.630	0.890

PPD: pocket probing depth

Table S3. Comparison of distal PPD values between groups over time.

Time point	Mini-Labrida	Regular-Labrida	Mini-EMS	Regular-EMS	Test statistic	p-value
Baseline	4.0 (2–6)	4.0 (2–5)	4.0 (1–5)	4.5 (2–5)	2.893	0.408
2 weeks	3.0 (2–4)	2.0 (2–3)	4.0 (1–5)	4.5 (2–5)	18.538	<0.001*
4 weeks	2.5 (2–4)	2.0 (1–3)	4.0 (1–5)	4.5 (2–5)	18.792	<0.001*

12 weeks	2.5 (2–4)	2.0 (1–3)	2.5 (1–4)	3.0 (2–4)	5.944	0.114
24 weeks	2.5 (2–4)	2.0 (1–3)	2.5 (1–4)	3.0 (2–3)	3.966	0.265

PPD: pocket probing depth

Table S4. Comparison of lingual/palatal PPD values between groups over time.

Time point	Mini-Labrida	Regular-Labrida	Mini-EMS	Regular-EMS	Test statistic	p-value
Baseline	2.0 (2–4)	2.0 (2–5)	2.0 (1–3)	2.0 (1–5)	3.613	0.306
2 weeks	2.0 (1–3)	2.0 (1–3)	2.0 (1–3)	2.0 (1–5)	2.889	0.409
4 weeks	2.0 (1–2)	2.0 (1–3)	2.0 (1–3)	2.0 (1–5)	4.430	0.219
12 weeks	2.0 (1–2)	2.0 (1–3)	2.0 (1–2)	2.0 (1–4)	1.802	0.615
24 weeks	2.0 (1–2)	2.0 (1–3)	2.0 (1–2)	2.0 (1–3)	1.737	0.629

PPD: pocket probing depth

Table S5. Comparison of plaque index and bleeding on probing between groups over time.

Time point	PI OCB	PI AAD	p-value (PI)	BoP OCB	BoP AAD	p-value (BoP)
Baseline	1.0 (1–2)	2.0 (1–3)	0.107	70%	68%	0.438
2 weeks	1.0 (0–2)	2.0 (1–3)	0.002*	56%	40%	0.002*
4 weeks	1.0 (0–2)	2.0 (0–2)	0.003*	60%	30%	0.011*
12 weeks	1.0 (0–2)	2.0 (0–2)	0.002*	66%	57%	0.092
24 weeks	1.0 (0–2)	2.0 (0–2)	0.002*	66%	50%	0.068

*Values presented as median (IQR) for PI and % of sites for BoP. p-values from Kruskal–Wallis test (PI) and Fisher's Exact Test with Monte Carlo correction (BoP). OCB: oscillating chitosan brush; PPD: pocket probing depth; BoP: bleeding on probing, AAD: air-abrasive device, PI: plaque index
